# Reinforcing the new diagnosis of Complex Post-Traumatic Stress disorder (CPTSD) of ICD-11: exploring the clinical outcomes in youth exposed to complex trauma

**DOI:** 10.1192/j.eurpsy.2022.1139

**Published:** 2022-09-01

**Authors:** L. Marques Feixa, S. Romero, J. Moya-Higueras, P. Santamarina-Pérez, J. March-Llanes, M.J. Muñoz, I. Zorrilla, M. Rapado-Castro, H. Blasco-Fontecilla, E. Anglada, M. Ramirez, L. Fañanas

**Affiliations:** 1 University of Barcelona, Evolutionary Biology, Ecology And Environmental Sciences, Barcelona, Spain; 2 Hospital Clínic of Barcelona, Department Of Child And Adolescent Psychiatry And Psychology, Barcelona, Spain; 3 Universitat de Lleida, Department Of Psychology, Lleida, Spain; 4 Hospital Benito Menni, Adolescent Crisis Unit, Sant Boi de Llobregat, Spain; 5 Hospital Santiago Apostol, Department Of Psychiatry, Vitoria-Gasteiz , Spain; 6 Hospital General Universitario Gregorio Marañón, Department Of Child And Adolescent Psychiatry, Madrid, Spain; 7 Hospital Puerta de Hierro, Department Of Child And Adolescent Psychiatry, Majadahona, Spain; 8 Fundació Orienta Gavà, Hospital For Adolescents, Gava, Spain; 9 Galdakao Mental Health Services, Child And Adolescent Mental Health, Galdakao, Spain

**Keywords:** Maltreatment, complex trauma, youth, CPTSD

## Abstract

**Introduction:**

Youth exposed to complex trauma (CT) show an increased risk of psychiatric morbidity, including a wide range of psychiatric disorders. However, to date, there is no specific diagnosis in the DSM-5 that capture the clinical complexity of these patients. Properly, the last version of the ICD-11 includes a diagnosis termed Complex Post-Traumatic Stress Disorder (CPTSD), which considers the pattern of post-traumatic stress symptoms, plus life-impairing disturbances in self-organization (emotion dysregulation, negative self-concept and interpersonal problems). Clinical research about CPTSD, especially in younger population, is still limited.

**Objectives:**

To explore the symptomatology of CPTSD in a sample of youth exposed to CT and its association with worse clinical outcomes.

**Methods:**

187 youth aged 7 to 17 years participated in the EPI_young_stress_project (116 with current psychiatric disorder and 71 healthy controls). CT was evaluated following the TASSCV criteria. To identify CPTSD symptomatology, we performed an exploratory factor analysis including CBCL and TEIQue items. The global level of functioning was measured by CGAS.

**Results:**

Preliminary results pointed that youth exposed to CT showed greater internalizing (p<.001) and externalizing (p<.001) symptomatology. Regardless of their current primary diagnosis based on DSM-5, youth exposed to CT reported more CPTSD symptomatology (p<.001). Moreover, youth with CPTSD showed greater use of psychotropic drugs (p<.001), higher and longer hospitalizations (p=.002) and worse overall functioning (p<.001).

**Conclusions:**

The inclusion of the CPTSD in future versions of mental disorders manuals should increase the implementation of early specific trauma interventions, which may improve victims’ lives and reduce the risk of worse clinical outcomes.

**Disclosure:**

No significant relationships.

